# Anti-RBD IgG antibodies from endemic coronaviruses do not protect against the acquisition of SARS-CoV-2 infection among exposed uninfected individuals

**DOI:** 10.3389/fimmu.2024.1396603

**Published:** 2024-05-23

**Authors:** Flávia Lopes Adami, Mateus Vidigal de Castro, Bianca da Silva Almeida, Isabela Pazotti Daher, Márcio Massao Yamamoto, Keity Souza Santos, Mayana Zatz, Michel Satya Naslavsky, Daniela Santoro Rosa, Edecio Cunha-Neto, Vivian Leite de Oliveira, Jorge Kalil, Silvia Beatriz Boscardin

**Affiliations:** ^1^ Departamento de Parasitologia, Instituto de Ciências Biomédicas, Universidade de São Paulo, São Paulo, Brazil; ^2^ Centro de Estudos do Genoma Humano e Células Tronco, Universidade de São Paulo, São Paulo, Brazil; ^3^ Departamento de Genética e Biologia Evolutiva, Instituto de Biociências, Universidade de São Paulo, São Paulo, Brazil; ^4^ Laboratório de Imunologia, LIM19, Instituto do Coração (InCor), Hospital das Clínicas da Faculdade de Medicina da Universidade de São Paulo (HCFMUSP), São Paulo, Brazil; ^5^ Departamento de Clínica Médica, Disciplina de Alergia e Imunologia Clínica, Faculdade de Medicina da Universidade de São Paulo (FMUSP), São Paulo, SP, Brazil; ^6^ Instituto de Investigação em Imunologia-Instituto Nacional de Ciências e Tecnologia (iii-INCT), São Paulo, Brazil; ^7^ Departamento de Microbiologia, Imunologia e Parasitologia, Disciplina de Imunologia, Universidade Federal de São Paulo (UNIFESP), São Paulo, Brazil

**Keywords:** seasonal coronavirus, COVID-19, humoral immunity, cross-reactivity, RBD protein

## Abstract

**Background:**

The Coronaviridae family comprises seven viruses known to infect humans, classified into alphacoronaviruses (HCoV-229E and HCoV-NL63) and betacoronaviruses (HCoV-OC43 and HCoV-HKU1), which are considered endemic. Additionally, it includes SARS-CoV (severe acute respiratory syndrome), MERS-CoV (Middle East respiratory syndrome), and the novel coronavirus SARS-CoV-2, responsible for COVID-19. SARS-CoV-2 induces severe respiratory complications, particularly in the elderly, immunocompromised individuals and those with underlying diseases. An essential question since the onset of the COVID-19 pandemic has been to determine whether prior exposure to seasonal coronaviruses influences immunity or protection against SARS-CoV-2.

**Methods:**

In this study, we investigated a cohort of 47 couples (N=94), where one partner tested positive for SARS-CoV-2 infection via real-time PCR while the other remained negative. Plasma samples, collected at least 30 days post-PCR reaction, were assessed using indirect ELISA and competition assays to measure specific antibodies against the receptor-binding domain (RBD) portion of the Spike (S) protein from SARS-CoV-2, HCoV-229E, HCoV-NL63, HCoV-OC43, and HCoV-HKU1.

**Results:**

IgG antibody levels against the four endemic coronavirus RBD proteins were similar between the PCR-positive and PCR-negative individuals, suggesting that IgG against endemic coronavirus RBD regions was not associated with protection from infection. Moreover, we found no significant IgG antibody cross-reactivity between endemic coronaviruses and SARS-CoV-2 RBDs.

**Conclusions:**

Taken together, results suggest that anti-RBD antibodies induced by a previous infection with endemic HCoVs do not protect against acquisition of COVID-19 among exposed uninfected individuals.

## 1 Introduction

Human coronaviruses (HCoVs) are zoonotic viruses of the Coronaviridae family that can cause severe respiratory infections (1)and rank as the second cause of the common cold after rhinoviruses (2). There are currently seven known human-infecting coronaviruses: seasonal alphacoronaviruses HCoV-229E and HCoV-NL63, betacoronaviruses HCoV-OC43 and HCoV-HKU1, and the emergent severe acute respiratory syndrome coronavirus (SARS-CoV), Middle East respiratory syndrome coronavirus (MERS-CoV), and the novel severe acute respiratory syndrome coronavirus 2 (SARS-CoV-2) (3). Typically, seasonal or common coronaviruses cause mild upper-respiratory tract infections in immunocompetent individuals, although severe lower-respiratory tract disease can affect children, the elderly, and immunocompromised individuals (4). HCoV-229E (5) and HCoV-OC43 (6) were isolated over 50 years ago, while HCoV-NL63 (7) and HCoV-HKU1 (8) were identified after the 2002 SARS-CoV outbreak in China. These viruses are endemic, contributing to an estimated 15–30% of respiratory tract infections each year (4). However, the real clinical importance of these viruses remains undefined due to conflicting data in the literature and the lack of studies specially designed to directly address their infection prevalence.

HCoV-229E was the first coronavirus to be discovered in 1966 (5), belongs to the Duvinacovirus subgenus, and causes common colds in healthy individuals and susceptible populations like children and the elderly. Despite its association to common colds, HCoV-229E has been detected in severe infections of the lower-respiratory tract among healthy adults with no comorbidities, leading to cases of pneumonia or bronchiolitis. The precise reasons behind the varying clinical manifestations observed in different patient groups remain unclear (9, 10). HCoV-OC43, discovered in 1967 (6), is the most prevalent coronavirus related to infections and was the second coronavirus identified. Named with the prefix ‘OC’ from organ culture, it belongs to the Embecovirus subgenus and can infect both humans and cattle (11). Discovered in the Netherlands in 2004 (7), HCoV-NL63 is directly associated with common cold manifestations but can also lead to more serious infections of the lower-respiratory tract. Similar to the virus causing COVID-19 (SARS-CoV-2), HCoV-NL63 is the only seasonal coronavirus known to use the human angiotensin-converting enzyme 2 (ACE2) as cell penetration receptor, although studies suggest that the Spike (S) protein from HCoV-NL63 has a weaker interaction with human ACE2 than SARS-CoV-2 (12, 13). HCoV-HKU1 was the last seasonal coronavirus to be discovered in Hong Kong in 2005 (8), and it seems to have originated from infected mice. Among all seasonal coronaviruses, HCoV-HKU1 infection is associated with more severe symptoms such as chills, tonsillar hypertrophy and febrile seizures. Infections with this virus are usually self-limiting, with only two reported pneumonia-related deaths in patients with serious underlying conditions like cancer (8, 14).

SARS-CoV-2 was identified in Wuhan, Hubei province, China, in individuals exposed at a seafood market that also commercialized live animals, suggesting zoonotic transmission. However, until now, it is not known how the virus spilled over from its original host to the market and, consequently, to people. SARS-CoV-2 infection causes COVID-19, declared a global public health emergency, displaying symptoms ranging from mild colds (80% of symptomatic cases) to more severe manifestations (5–10% of cases) such as pneumonia, respiratory failure, heart failure, sepsis and multi-organ failure, as well as, asymptomatic cases (15).

The main protein of coronaviruses, Spike (S), is a glycoprotein of approximately 180 kilodaltons (kDa), located on the viral surface. Its sequence encodes a signal peptide at the N-terminus and the S1 and S2 subunits, responsible for receptor binding and membrane fusion, respectively (16). Mutations in the gene encoding Spike enable its adaptation to new tissues and hosts (17–19). Given its role in virus entry into host cells, the S protein is the primary target for neutralizing antibodies and a focus for therapeutic and vaccine strategies. Potent neutralizing antibodies usually target the receptor-binding domain (RBD) located in the S1 subunit, blocking viral entry by preventing the interaction of the S1 subunit with the ACE2 receptor (20, 21).

Cross-immunity occurs when an immune response triggered by one pathogen confers partial or complete protection against a related pathogen, relying on common antigens shared by both pathogens. Cross-reactivity to seasonal coronaviruses may be significant for COVID-19, as studies indicate the presence of SARS-CoV-2 specific CD4^+^ T cells in individuals not previously exposed. Thus, cross-reactivity in T cell recognition is plausible, since there are homologous sequences among the different types of human-infecting coronaviruses (22).

However, while extensive exploration has been conducted on the degree of antibody cross-reactivity between endemic HCoVs and SARS-CoV-2, findings remain controversial. A study reported 2.3% seropositivity (53 out of 1938 samples) in immunoassays against nucleoprotein (NP) and RBD protein in individuals likely unexposed to the virus, suggesting potential cross-reactivity against SARS-CoV-2 (23). Other studies revealed pre-existing antibodies against SARS-CoV-2 in unexposed individuals directed specifically to the S2 subunit of the Spike protein, but not the S1 subunit which includes the RBD (24), lacking neutralizing or protective activity against SARS-CoV-2 infection (24–26). In addition, cross-reactive antibodies potentially induced by previous endemic HCoV infections were also detected against ORF1 and, to a lesser extent, Spike and NP (27).

Evidence also suggests that an immune response against seasonal coronaviruses might correlate to a better prognosis in COVID-19 progression (28). Moreover, previous responses to endemic HCoVs might influence the functionality of the anti-SARS-CoV-2 antibody repertoire responses (29, 30).

In order to evaluate the endemic HCoVs’ anti-RBD IgG response profile, its association with COVID-19 acquisition, and the cross-reactivity of endemic HCoV RBD with SARS-CoV-2 RBD, we tested plasma samples from couples living together, where one individual acquired COVID-19, while the other remained uninfected despite exposure in the same household. Our results showed that anti-RBD IgG responses to endemic HCoVs did not predict protection against infection, discarding the potential cross-reactive effect from previous endemic coronavirus exposure on the antibody repertoire against SARS-CoV-2 infections.

## 2 Materials and methods

### 2.1 Volunteers’ recruitment, blood collection, and sample processing

For this study, we selected a cohort of 47 Brazilian couples who showed discordant results in real-time PCR tests for SARS-CoV-2 detection, during the first wave of COVID-19 in Brazil in 2020, as detailed in **Supplementary Table 1**. In each selected couple, one partner tested positive for COVID-19 via PCR, while the other tested negative. The infected partner exhibited symptoms of COVID-19, while the other partner remained uninfected (as confirmed by a negative PCR result), despite sharing the same living space and sleeping arrangements throughout the period of infection. The couples did not maintain social distance during the course of the illness, did not wear masks and did not take any protective measures at home. The members of each couple were of similar age (between 24 and 79 years, with an average age of 44.4 years) and had access to the same health insurance plan. Also, individuals with pre-existing diseases and/or comorbidities that could influence the course of the disease were not included in this cohort.

Members infected within the cohort were classified into subgroups according to their COVID-19 clinical conditions, based on the severity scales proposed by the World Health Organization (WHO-2019-nCoV-clinical-2020.5) and elaborated upon by Gandhi et al. (31). The classifications are as follows: Mild illness: characterized by the presence of common symptoms such as fever, cough, and changes in smell (anosmia) and taste (dysgeusia), but not shortness of breath (dyspnea). No hospitalization is required; Moderate illness: defined by the presence of common symptoms, including dyspnea, and either clinical or radiographic evidence of lower respiratory tract disease, but without hypoxemia (blood oxygen saturation of 94% or higher). Hospitalization may be warranted. In this study, none of the participants were hospitalized, and all infected participants recovered without any complications or sequelae.

Blood samples were collected in vacutainer tubes containing EDTA (BD Biosciences) from both partners at least one month after the initial illness (to detect SARS-CoV-2 antibodies) prior to the availability of COVID-19 vaccines in Brazil, and before the appearance of new SARS-CoV-2 variants (between June and October 2020). To ensure that the partners who tested negative had not contracted the virus asymptomatically, we conducted serological tests to confirm their seronegative status. Plasma was separated by centrifuging the samples at 2000 x g for 10 minutes at room temperature, performed within 30 minutes of venipuncture. Subsequently, the supernatant was aliquoted into 1.5 mL cryovials (Corning^®^, USA). These samples were then stored at -80°C until further analysis.

Each couple had their sample for real-time PCR testing collected on the same day the partner was confirmed positive, and his/her plasma sample was collected on the same day as the positive partner was collected.

### 2.2 Production of the RBD from HCoVs and SARS-CoV-2

Plasmids containing nucleic acid sequences encoding the RBD protein sequences of four seasonal human coronaviruses (HCoV-OC43, HCoV-NL63, HCoV-229E, and HCoV-HKU1) were kindly provided by Dr. Aravinda M. de Silva (University of North Carolina School of Medicine, Chapel Hill, USA) and are described in (32). Additionally, the RBD protein sequence from SARS-CoV-2 Wuhan Hu-1 strain was kindly provided by Dr. Florian Krammer (Icahn School of Medicine at Mount Sinai, New York, USA) and is described in (33).

The plasmids were transformed by heat shock into TOP10 competent *Escherichia coli* bacteria (ThermoFisher Scientific). A single colony was cultured for 16–18 hours in 200 mL of LB medium (Merck) supplemented with 100 μg/mL of ampicillin. Plasmid DNA extraction was performed using the PureLink™ HiPure Plasmid MaxiPrep kit (ThermoFisher Scientific) exactly as advised by the manufacturer. The concentrations of the purified plasmids were determined by spectrophotometry (NanoDrop 2000, ThermoFisher Scientific), and their integrity was analyzed using 0.8% agarose gels.

Expi293F™ cells (ThermoFisher Scientific) were grown at a concentration of 1–3x10^6^ cells/mL. The cells were thawed in a water bath and then fed into a flask containing 30 mL of pre-warmed Expi293™ expression medium (ThermoFisher Scientific). The cells were diluted every 2–3 days, depending on the density found, and at least 3 passages were made with the addition of a new medium, before transfection. Transfections were performed exactly as described in (34).

### 2.3 Purification of RBD Proteins by affinity chromatography

On day 5, transfected cell cultures were harvested after the addition of 100 mM of phenylmethylsulfonyl fluoride (PMSF, ThermoFisher Scientific). After centrifugation at 3.000 xg for 20 min, the supernatants were collected and an equivalent volume of cold 1x PBS (Phosphate Buffered Saline) was added. Five mL plastic columns were set up with 1 mL of HisPur™ Ni-NTA resin (ThermoFisher Scientific) for each 100 mL of culture supernatant. After two washes with 5 mL of cold 1x PBS, each column was adapted to a peristaltic pump (Mini-Peristaltic Pump II, Harvard Apparatus), and the cold supernatant was passed slowly twice. Subsequently, the columns were washed with 100 mL of cold 1x PBS containing 5mM imidazole (Merck). Elution was carried out initially with 50 mL of cold 1x PBS containing 25 mM imidazole followed by 10 mL of cold PBS 1x containing 250 mM of imidazole, collected in 1 mL-fractions. Protein presence in each fraction was evaluated using Bradford reagent (ThermoFisher Scientific). Protein-containing tubes were pooled together and dialyzed against cold 1x PBS to remove imidazole. Protein concentration was quantified by spectrophotometry (NanoDrop 2000, ThermoFisher Scientific), and protein integrity was confirmed via 12% polyacrylamide gel electrophoresis and Coomassie blue staining (BioRad) (**Supplementary Figure 1**).

### 2.4 Indirect ELISA

To assess plasma reactivity against RBD proteins from seasonal HCoVs and SARS-CoV-2, and against the nucleoprotein (NP, kindly provided by Dr. Ricardo T. Gazzinelli, Federal University of Minas Gerais, Brazil (35)), an Enzyme Linked Immunosorbent Assay (ELISA) was performed. High binding 96-well ELISA plates (Costar) were incubated with 100 ng/well of each RBD or NP protein diluted in 1x PBS at room temperature (RT) for 18 hours. The plates were then washed 3x with 1x PBS+0.02% Tween (Synth, PBS-T). Blocking was performed for 1 hour at RT with 150 μL/well of PBS-T containing 1% bovine serum albumin (BSA, Merck) and 5% powdered skim milk (Nestle). After 3 more washes with PBS-T, plasma samples were diluted 1:100, in duplicates, in 100 μL/well of PBS-T containing 0.25% BSA and 5% powdered skim milk, and incubated at 37 °C for 2 hours. Following three additional washes with PBS-T, plates were incubated with 50 µL/well of a secondary anti-human IgG-HRP antibody (1:15,000, KPL) for 1-hour incubation at RT. After three washes with PBS-T, plates were developed using a solution containing 1mg/mL ortho-phenylenediamine dihydrochloride (OPD, Amresco), 0.2 M sodium phosphate and 0.1 M citric acid (pH 4.7) plus 30% H_2_O_2_. The reaction was stopped with 50 µL of 4N H_2_SO_4_ (Merck) solution after 15 minutes, and plates were read at a wavelength of 492 nm using the BioTek ELx800 reader (Biotek).

### 2.5 Competition ELISA

To examine the cross-reactivity of antibodies present in the plasma of each patient, competition ELISA assays were conducted. Plasma from each patient was adsorbed or not with 20 µg/mL of each recombinant RBD for 2 hours at 37°C. Then, the plasmas were diluted 1:100, in duplicates, in 100 μL/well of PBS-T containing 0.25% BSA and 5% powdered skim milk, and transferred to high binding 96-well ELISA plates (Costar) containing 100 ng/well of each RBD (previously diluted in 1x PBS at RT for 18 hours, and washed 3x with 1x PBS-T). Diluted plasmas were then incubated at 37 °C for 2 hours. Following three additional washes with PBS-T, plates were incubated with 50 µL/well of a secondary anti-human IgG-HRP antibody (1:15,000, KPL) for 1-hour incubation at RT. After three washes with PBS-T, plates were developed using a solution containing 1mg/mL ortho-phenylenediamine dihydrochloride (OPD, Amresco), 0.2 M sodium phosphate and 0.1 M citric acid (pH 4.7) plus 30% H_2_O_2_. The reaction was stopped with 50 µL of 4N H_2_SO_4_ (Merck) solution after 15 minutes, and plates were read at a wavelength of 492 nm using the BioTek ELx800 reader (Biotek). The O.D. readings obtained for each duplicate without or with RBD adsorption were recorded. The ratio was obtained by dividing the mean values of the O.D. readings obtained without and with RBD adsorption.

### 2.6 Statistical data analysis

Normality testing was performed using the D’Agostino & Pearson test. For data not passing normality test, non-parametric Kruskal-Wallis test followed by Dunn’s multiple comparisons test were utilized. Non-parametric Mann-Whitney test was used when two groups were compared. One-way ANOVA for repetitive measures was used for data passing the normality test. The GraphPad Prism 9 software was used for data analysis and significance was set at P < 0.05.

## 3 Results

### 3.1 Plasma reactivity to SARS-CoV-2 RBD and nucleocapsid protein

**Figure 1:**
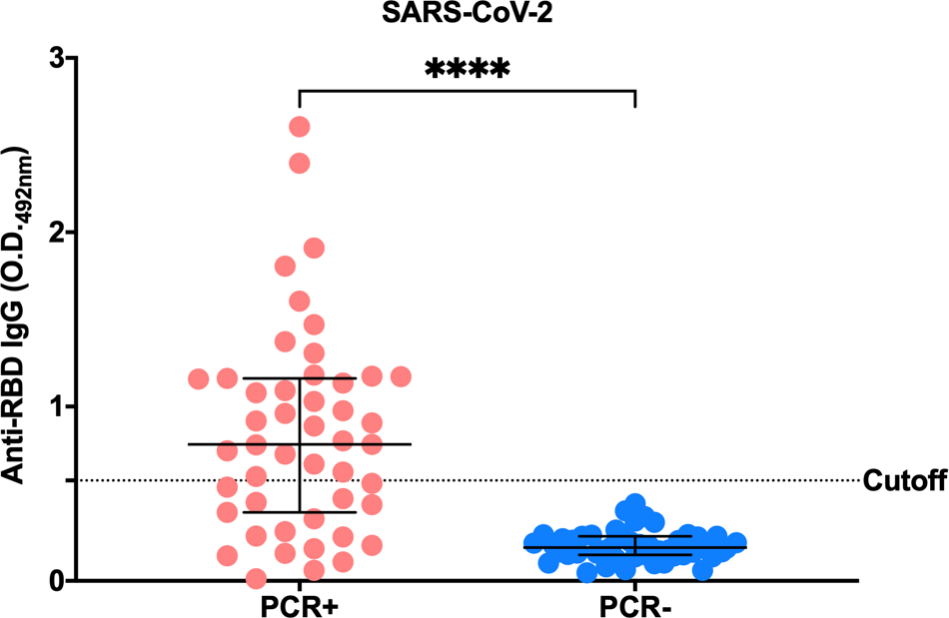
SARS-CoV-2 anti-RBD IgG serology profile. Indirect ELISA performed in order to identify the IgG response profile of PCR+ or PCR- individuals against the RBD portion of SARS-CoV-2 Spike protein. Plasma samples were used at a concentration of 1:100 and recombinant RBD at 2 µg/ml (100 ng/well). The test was read at a wavelength of 492 nm. The horizontal black lines represent the medians ± interquartile intervals. Cutoff = Average O.D. of known negative individuals (pre-pandemic samples) + 3 standard deviations. Mann-Whitney test, ****p<0.0001.

Initially, we tested plasma reactivity against SARS-CoV-2 Wild-type (Wuhan-Hu-1) strain by ELISA (**Figure 1**). All PCR-negative (PCR-) individuals presented reactivities below the set cut-off (calculated using 8 pre-pandemic plasma samples plus 3 standard deviations, cutoff= 0.576). Notably, 17 out of 47 PCR-positive (PCR+) individuals failed to seroconvert IgG antibodies against SARS-CoV-2 RBD 30 days after infection, despite the confirmation of infection by real-time PCR. The detailed data are presented in **Supplementary Table 1**. Although 30 days is generally sufficient for most individuals to develop detectable IgG antibodies, there are significant individual variations in the immune response. These individuals may have generated antibodies against distinct components of the virus, besides the RBD. To test if this was the case, we conducted serological testing for the nucleocapsid protein (NP). Among the 17 individuals without antibodies against SARS-CoV-2 RBD, 9 of them exhibited antibodies to SARS-CoV-2 NP (**Supplementary Table 1**). Notably, all seven volunteers diagnosed with moderate COVID-19 exhibited detectable levels of SARS-CoV-2 antibodies, targeting the receptor binding domain (RBD) or the nucleocapsid protein (NP).

### 3.2 Plasma reactivity to HCoV RBDs

**Figure 2:**
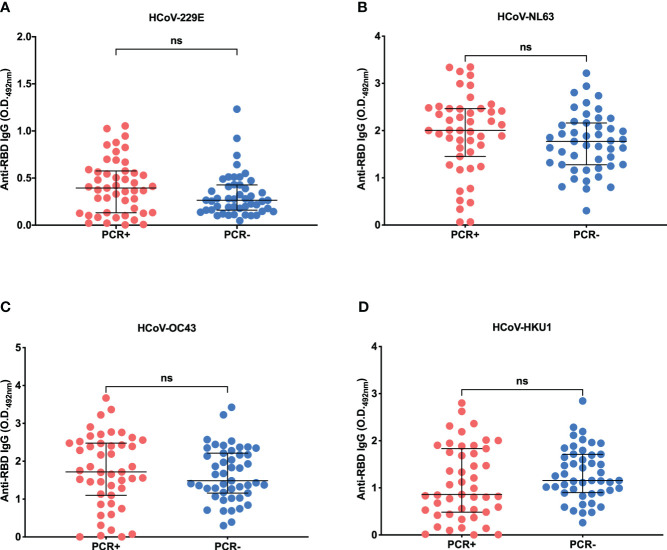
Anti-RBD IgG serology profile of HCoV-229E, HCoV-OC43, HCoV-NL63 and HCoV HKU1. IgG response profile of PCR+ or PCR- individuals for SARS-CoV-2 against RBD proteins derived from alphacoronaviruses HCoV-229E **(A)** and HCoV-NL63 **(B)**, and betacoronaviruses HCoV-OC43 **(C)** and HCoV-HKU1 **(D)**. For this assay, the subjects’ plasma was used at a concentration of 1:100, and the recombinant RBD in 2 µg/ml (100 ng/well). The test was read in the wavelength of 492 nm and each graph shows the O.D. obtained for the PCR+ (red) or PCR- (blue) individuals for SARS-CoV-2. The horizontal black lines represent the medians ± interquartile intervals. Mann-Whitney test. ns, not significant.

We further investigated plasma reactivity against a panel of HCoV RBDs produced in eukaryotic cells (**Supplementary Figure 1**). **Figure 2** shows the reactivity of PCR-positive and negative individuals’ plasmas against a panel of recombinant HCoV RBD proteins (HCoV-229E, HCoV-NL63, HCoV-OC43 and HCoV-HKU1). The analysis revealed robust IgG-specific antibody responses to HCoVs among most individuals in the cohort, irrespective of COVID-19 status, displaying relatively high O.D. values (**Figures 2B-D**), except for HCoV-229E which showed lower reactivity compared to the other HCoVs (**Figure 2A**). However, more importantly, no statistically significant differences were detected when comparing anti-RBD responses between SARS-CoV-2 PCR-positive and negative individuals (p=0.1054 for HCoV-229E, p=0.1022 for HCoV-NL63, p=0.3347 for HCoV-OC43 and p=0.1113 for HCoV-HKU1). Additionally, when dividing the individuals into groups based on their PCR results and gender, no statistical differences were observed (**Supplementary Figures 2A-D**). Regarding the reactivity to SARS-CoV-2, both PCR-positive males and females exhibited statistically significant differences in comparison to PCR-negative subjects, but not between PCR-positive or PCR-negative individuals (**Supplementary Figure 2E**).

### 3.3 Competition ELISAs to detect anti-HCoV RBD-specific antibodies

**Figure 3:**
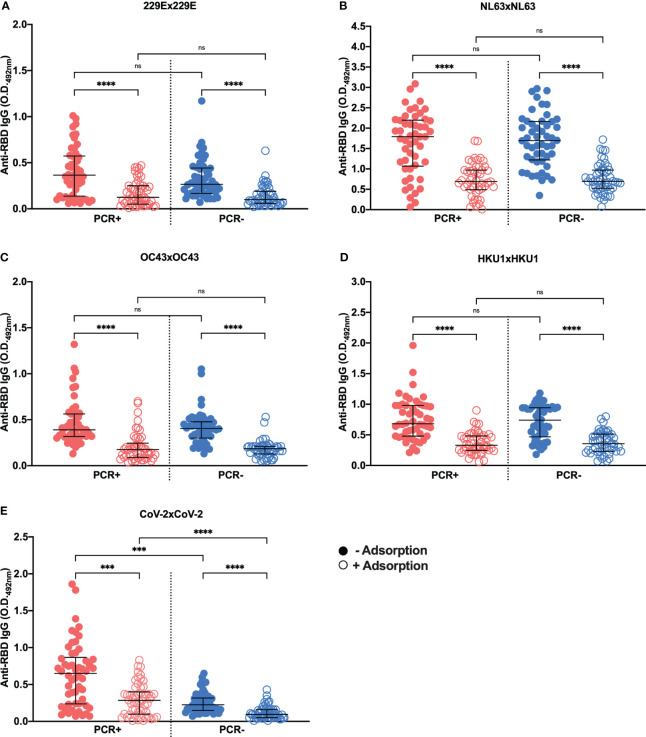
Cross-reactivity testing using SARS-CoV-2 PCR-positive (PCR+) and negative (PCR-) samples adsorbed or not against each RBD protein. Graphs show ELISA assays with samples of SARS-CoV-2 PCR+ or PCR- individuals against RBD proteins from HCoV-229E **(A)**, HCoV-NL63 **(B)**, HCoV-OC43 **(C)**, HCoV-HKU1 **(D)** and SARS-CoV-2 **(E)** adsorbed (open circles) or not (filled circles) to their respective proteins. Plasma from the subjects was used at a concentration of 1:500 and the recombinant RBD for coating diluted to 2 µg/mL (100 ng/well) and for adsorption at 20 µg/mL. The assay read-out was performed at a wavelength of 492 nm. The graphs show O.D. values for SARS-CoV-2 PCR+ (red) or PCR- (blue) individuals, with and without adsorption with the respective RBD protein. The horizontal black lines represent the medians ± interquartile intervals. Kruskal-Wallis followed by the Dunn’s test. ***p<0.001 and ****p<0.0001; ns, not significant.

The lack of negative controls, i.e. plasma samples that were known to be negative for each HCoV, did not allow us to calculate a cut-off for each HCoV RBD protein, as we did for SARS-CoV-2 in **Figure 1**. To overcome this limitation and indeed check if we could detect anti-HCoV RBD specific antibodies, plasma samples were pre-incubated with each HCoV RBD, followed by testing on ELISA plates containing the same HCoV RBD. A reduction in response to each HCoV RBD was observed upon previous incubation with the respective HCoV RBD (**Figure 3**). However, no differences were detected between SARS-CoV-2 PCR-positive and negative individuals regarding plasma reactivity to HCoV-229E (**Figure 3A**), HCoV-NL63 (**Figure 3B**), HCoV-OC43 (**Figure 3C**) or HCoV-HKU1 (**Figure 3D**), in both non-adsorbed or previously adsorbed samples. As expected, significant differences were detected when comparing PCR-positive and negative plasma samples tested against SARS-CoV-2 RBD (**Figure 3E**), in line with the clinical and molecular diagnostics.

### 3.4 Adsorption of plasma samples and evaluation of cross reactivity against each previously tested RBD

**Figure 4:**
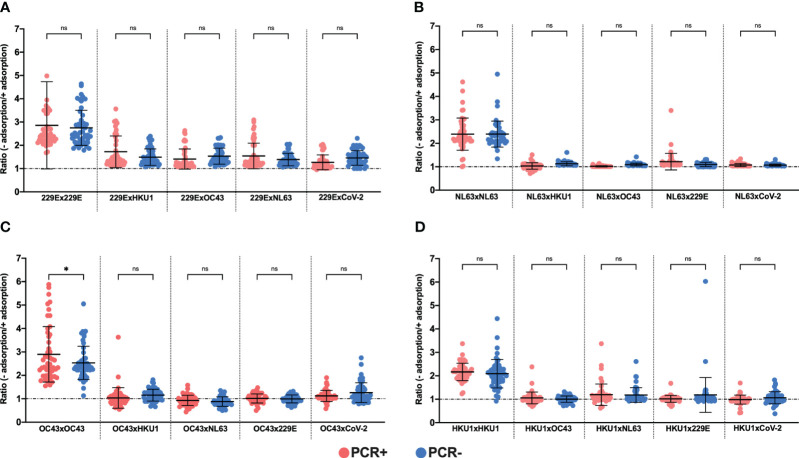
Ratio between O.D. values of plasma samples not adsorbed and adsorbed to recombinants RBDs of seasonal coronavirus. To assess cross-reactivity between proteins seasonal coronavirus RBDs were performed in competition ELISA assays, using 100 plasma samples from PCR+ (red) and PCR- (blue) individuals to COVID-19. The charts show the data normalized from non-adsorbed and adsorbed plasma samples against RBDs proteins from HCOV-229E **(A)**, HCOV-NL63 **(B)**, HCOV-OC43 **(C)** and HCOV-HKU1 **(D)**. The individual plasma was used in a title of 1:500 and the recombinant RBD for the coating was diluted in 2 µg/mL (100 ng/well) and for adsorption at 20 µg/mL. The assay reading was performed at a wavelength of 492 nm and each symbol shows the value of the ratio between the O.D. values without adsorption/with adsorption. The horizontal black lines represent the mean ± SD. One-way ANOVA for repetitive measures. *p<0.01; ns, not significant.

We proceeded to adsorb or not the plasma samples from PCR-positive and negative individuals to each HCoVs or SARS-CoV-2 RBD protein and tested them against each HCoV RBD in competition ELISA assays (**Figure 4**). The normalization of the data involved calculating a ratio of O.D. values obtained without adsorption to those obtained after adsorption. Notably, no statistical differences were found when we compared the ratios of PCR-positive and negative samples for HCoV-229E or the other HCoVs (**Figures 4A-D**), except a small difference observed for PCR-positive and negative individuals adsorbed against HCoV-OC43 and tested to itself (**Figure 4C**). It is important to mention that when the samples were adsorbed to a specific protein and subsequently tested with the same protein, we expected an increase in the ratios. As previously observed in **Figure 3**, adsorption resulted in the removal of the anti-RBD specific antibodies, leading to lower O.D. values when compared to non-adsorbed samples. This is precisely what we observed when the plasma samples from PCR-positive and negative individuals were adsorbed and tested against the same protein.

## 4 Discussion

In this study, we conducted a comparative analysis of antibody responses to the RBD from the four endemic coronaviruses - HCoV-OC43, HCoV-NL63, HCoV-229E and HCoV-HKU1 – as well as the SARS-CoV-2 RBD. The focus was on individuals in intimate relationships, specifically couples (n=47), who either had symptomatic COVID-19 (SARS-CoV-2 PCR positive) or had not (SARS-CoV-2 PCR negative spouses). Among the infected participants, the majority exhibited mild symptoms (n=40) and were predominantly male, supporting other studies that suggested men were more prone to symptomatic presentations of COVID-19 than females during the first outbreak (2020) of the disease (36, 37). Here, our results indicate that both SARS-CoV-2 infected and uninfected individuals exhibit similar levels of IgG antibodies against the RBD portion of endemic coronaviruses. Furthermore, IgG antibodies against the RBD of different endemic coronaviruses did not show cross-reactivity with the SARS-CoV-2 RBD or with each other.

Our initial results revealed that not every individual who tested positive for SARS-CoV2 infection via PCR (17 out of 47) underwent RBD seroconversion within 30 days following the PCR positive test. In some cases, seroconversion may occur later than expected. Alternatively, these individuals might have developed antibodies targeting different viral components, such as the nucleocapsid protein (NP), a highly immunogenic protein also used in the serological diagnosis of SARS-CoV-2 infection (35, 38). Among those 17 individuals showing reactivity to SARS-CoV-2 below the threshold, 9 had developed antibodies to the SARS-CoV-2 NP. The remaining 8 PCR+ participants who did not show seroconversion for either SARS-CoV-2 RBD or NP might require additional time post-infection to produce anti-RBD/NP antibodies or might be relying on different immune responses that do not involve antibody production. Instead, these mechanisms could include the activation of T cells (39). It is important to mention that further monitoring of seroconversion in these participants was not possible at subsequent time points, as all volunteers received vaccinations shortly after the initial blood sample collection.

Regarding IgG antibody responsiveness to endemic coronavirus RBDs, most individuals, regardless of SARS-CoV-2 PCR status, displayed relatively high O.D. values against RBDs of different HCoVs. This was corroborated by the results of IgG homologous competition ELISAs, indicating that the majority of individuals in the PCR+ and PCR- groups showed similar ratios prior exposure to all HCoVs. However, results on homologous adsorption with HCoV-OC43 RBD did reveal a difference between ratios of non-adsorbed/adsorbed plasmas observed in PCR+ and PCR- subjects. This was unexpected since it was not apparent when comparing O.D. values of HCoV-OC43 RBD between the PCR+ and PCR- groups. Currently, we do not have a clear explanation for this difference. This may suggest that patients with COVID-19 could have increased antibody levels against HCoV-OC43 RBD and therefore could have been more exposed, or more recently exposed to it. A previous report using samples from the United States noticed an increase in reactivity against S protein of HCoV-HKU1 among individuals who developed COVID-19 disease as compared to COVID-19 negative subjects (40). At any event, our interpretation is that anti-RBD antibodies acquired in a previous infection with endemic coronaviruses play no role in the non-acquisition of SARS-CoV-2 infection of the PCR negative partner, thus having no protective effect. This conclusion is supported by previous data, where no significant differences were observed in the reactivity of sera from COVID-19 patients compared to individuals from the pre-pandemic period for the S proteins of HCoV-229E, HCoV-OC43 and HCoV-NL63 (40). Taken together, these findings suggest that SARS-CoV-2 infection might not induce the expansion of B cell clones with RBD-specific memory exhibiting cross-reactivity to any of the HCoVs, possibly due to the limited amino acid similarity (19 to 21%) between the RBDs of HCoVs and SARS-CoV-2 (32).

In terms of cross-reactivity between endemic coronavirus RBDs and SARS-CoV-2 RBD, as measured by the heterologous competition RBD ELISA assays, no cross-reactivity was observed between endemic coronaviruses and SARS-CoV-2 RBDs, as well as no cross-reactivity among different endemic coronaviruses themselves, while homologous competition ELISAs showed the expected reduction of reactivity after absorption. It is worth noting that, as a control, we included the SARS-CoV-2 RBD, for which the homologous competition ELISA was validated.

These findings suggest that SARS-CoV-2 infection may not induce the expansion of memory B cell clones previously activated with endemic coronavirus RBDs, possibly due to limited amino acid homology between the RBDs of SARS-CoV-2 and those of HCoVs (32). However, it is critical to clarify that our findings do not suggest a complete absence of cross-reactivity between HCoVs and SARS-CoV-2. Indeed, several studies analyzing pre-pandemic serum or plasma samples have identified a significant fraction showing reactivity with the S protein of SARS-CoV-2 (24, 40–42). Notably, Majdoubi et al. showed that over 90% of non-infected adults exhibited antibody reactivity against the S protein, its RBD, its N-terminal domain (NTD), or the NP of SARS-CoV-2 (41). Conversely, others have shown that the cross-reactive antibody responses against the S protein were predominantly targeted to the S2 fragment, a region of the S protein that is significantly more conserved (24, 42). Moreover, Song et al. provided limited evidence for the existence of pre-pandemic cross-reactive serum antibodies against SARS-CoV-2. However, they detected pre-existing cross-reactive memory B cells that underwent further expansion upon SARS-CoV-2 infection (40). A cross-reactive peptide within the S2 fragment of the S protein was subsequently identified (43). Following SARS-CoV-2 infection and vaccination, a 2- to 4-fold increase in antibodies that bind to seasonal HCoVs was observed in sera, compared to those from pre-pandemic healthy donors, with the S2 fragment being the main target of cross-reactivity (44). More recently, broadly neutralizing monoclonal antibodies targeting not the RBD but the S2 fragment were also developed (45, 46). Thus, the absence of cross-reactivity between the RBDs of various endemic HCoVs and SARS-CoV-2 observed in our study is consistent with these previous findings.

It should also be noted that the major limitation of our study was that we used ELISA readings as the only read-out for our experiments. Nonetheless, competition assays and the absence of statistical differences in antibody responses between PCR-positive and negative individuals suggest that pre-existing antibodies against HCoV RBDs may not confer protection against SARS-CoV-2 infection.

## Data availability statement

The original contributions presented in the study are included in the article/**Supplementary Material**. Further inquiries can be directed to the corresponding author.

## Ethics statement

The study was approved by the Committee for Ethics in Research of the Institute of Biosciences at the University of São Paulo (CAAE 34786620.2.0000.5464) following the Declaration of Helsinki principles, ICH06 Good Clinical Practices, and Brazilian Health Regulatory Agency (ANVISA) resolution number 466 from 2012 that regulates research with humans. Participants were enrolled in the study between June 24 and October 1, 2020, during which time blood samples were collected. The researchers in this study also had access to demographic and clinical data of the participants, which was necessary to integrate these findings with the serological results. However, the data remained anonymized and confidential to anyone unauthorized outside of this study. All participants were >18 years old and provided written informed consent prior to participation.

## Author contributions

FA: Formal analysis, Investigation, Methodology, Validation, Visualization, Writing – original draft. MC: Data curation, Resources, Writing – review & editing. BA: Investigation, Validation, Writing – review & editing. ID: Investigation, Validation, Writing – review & editing. MY: Investigation, Validation, Writing – review & editing. KS: Writing – review & editing. MZ: Funding acquisition, Resources, Writing – review & editing. MN: Data curation, Resources, Writing – review & editing. DR: Conceptualization, Methodology, Writing – review & editing. EC-N: Funding acquisition, Writing – review & editing. VO: Methodology, Project administration, Resources, Validation, Visualization, Writing – review & editing. JK: Funding acquisition, Resources, Writing – review & editing. SB: Conceptualization, Formal analysis, Funding acquisition, Methodology, Resources, Supervision, Validation, Visualization, Writing – review & editing.
