# Corrigendum: Anti-RBD IgG antibodies from endemic coronaviruses do not protect against the acquisition of SARS-CoV-2 infection among exposed uninfected individuals

**DOI:** 10.3389/fimmu.2025.1598312

**Published:** 2025-04-11

**Authors:** Flávia Lopes Adami, Mateus Vidigal de Castro, Bianca da Silva Almeida, Isabela Pazotti Daher, Márcio Massao Yamamoto, Keity Souza Santos, Mayana Zatz, Michel Satya Naslavsky, Daniela Santoro Rosa, Edecio Cunha-Neto, Vivian Leite de Oliveira, Jorge Kalil, Silvia Beatriz Boscardin

**Affiliations:** ^1^ Departamento de Parasitologia, Instituto de Ciências Biomédicas, Universidade de São Paulo, São Paulo, Brazil; ^2^ Centro de Estudos do Genoma Humano e Células Tronco, Universidade de São Paulo, São Paulo, Brazil; ^3^ Departamento de Genética e Biologia Evolutiva, Instituto de Biociências, Universidade de São Paulo, São Paulo, Brazil; ^4^ Laboratório de Imunologia, LIM19, Instituto do Coração (InCor), Hospital das Clínicas da Faculdade de Medicina da Universidade de São Paulo (HCFMUSP), São Paulo, Brazil; ^5^ Departamento de Clínica Médica, Disciplina de Alergia e Imunologia Clínica, Faculdade de Medicina da Universidade de São Paulo (FMUSP), São Paulo, SP, Brazil; ^6^ Instituto de Investigação em Imunologia-Instituto Nacional de Ciências e Tecnologia (iii-INCT), São Paulo, Brazil; ^7^ Departamento de Microbiologia, Imunologia e Parasitologia, Disciplina de Imunologia, Universidade Federal de São Paulo (UNIFESP), São Paulo, Brazil

**Keywords:** seasonal coronavirus, COVID-19, humoral immunity, cross-reactivity, RBD protein

In the published article, there was an error in **Supplementary Figure 2**. Specifically, the graph depicting HCoV-NL63, originally intended for panel B, was mistakenly replaced with the HCoV-OC43 graph, which was already present in panel D. This resulted in a duplication of the HCoV-OC43 data in both panels B and D. In addition, the HCoV-HKU1 and HCoV-OC43 graphs were misplaced in panels C and D, respectively. The correct order is HCoV-OC43 (C) and HCoV-HKU1 (D).

The authors apologize for this error and state that this does not change the scientific conclusions of the article in any way. The original article has been updated.

