# Supplementary material for: Anti-RBD IgG antibodies from endemic coronaviruses do not protect against the acquisition of SARS-CoV-2 infection among exposed uninfected individuals
Source: Front Immunol. 2024 May 23;15:1396603. doi: 10.3389/fimmu.2024.1396603 (PMC11153698; doi:10.3389/fimmu.2024.1396603)
Supplement: Supplementary file 1 [file DataSheet_1.pdf]

Supplementary Material

Supplementary Table 1. Cohort Data. Data from the cohort of real-time discordant couples used in the study: sample number, volunteer registration, gender (infected women: 35%; infected men: 65%), age (average age 46), PCR result (positive/ negative) and evolution of symptoms of COVID-19 (mild/moderate).

COHORT DATA								
**PCR discordant couples**	**Sample Number**	**Volunteer Registration**	**Gender**	**Age**	**PCR Result**	**Symptoms**	**Serology**	
							**RBD**	**NP**
**1**	**1**	C028680	M	77	Positive	Mild	Negative	Positive
	**2**	C028681	F	75	Negative	-		
**2**	**3**	C028688	F	46	Positive	Mild	Negative	Positive
	**4**	C028689	M	46	Negative	-		
**3**	**5**	C028690	M	37	Positive	Moderate	Positive	Positive
	**6**	C028691	F	35	Negative	-		
**4**	**7**	C028692	M	33	Positive	Moderate	Negative	Positive
	**8**	C028693	F	33	Negative	-		
**5**	**9**	C028696	F	37	Positive	Mild	Negative	Positive
	**10**	C028697	F	38	Negative	-		
**6**	**11**	C028708	M	41	Positive	Mild	Negative	Positive
	**12**	C028709	F	39	Negative	-		
**7**	**13**	C028720	M	65	Positive	Mild	Positive	Positive
	**14**	C028721	F	57	Negative	-		
**8**	**15**	C028724	M	38	Positive	Mild	**Negative**	**Negative**
	**16**	C028725	F	36	Negative	-		
**9**	**17**	C028730	M	44	Positive	Moderate	Positive	Positive
	**18**	C028731	F	42	Negative	-		
**10**	**19**	C028732	M	33	Positive	Mild	Positive	Positive
	**20**	C028733	F	35	Negative	-		
**11**	**21**	C028736	M	36	Positive	Mild	Positive	Positive
	**22**	C028737	F	35	Negative	-		
**12**	**23**	C028738	F	54	Positive	Mild	Positive	Positive
	**24**	C028739	M	54	Negative	-		
**13**	**25**	C028740	M	42	Positive	Mild	Positive	Positive

	**26**	C028741	F	48	Negative	-		
**14**	**27**	C028744	M	71	Positive	Mild	Positive	Positive
	**28**	C028745	F	68	Negative	-		
**15**	**29**	C028746	F	30	Positive	Mild	Positive	Positive
	**30**	C028747	F	29	Negative	-		
**16**	**31**	C028748	F	62	Positive	Moderate	Positive	Positive
	**32**	C028749	M	61	Negative	-		
**17**	**33**	C028750	M	52	Positive	Mild	**Negative**	**Negative**
	**34**	C028751	F	54	Negative	-		
**18**	**35**	C028752	M	56	Positive	Mild	Positive	Positive
	**36**	C028753	F	55	Negative	-		
**19**	**37**	C028757	M	54	Positive	Mild	Negative	Positive
	**38**	C028758	F	45	Negative	-		
**20**	**39**	C028764	M	70	Positive	Mild	**Negative**	**Negative**
	**40**	C028765	F	56	Negative	-		
**21**	**41**	C028766	F	34	Positive	Mild	Positive	Positive
	**42**	C028767	M	35	Negative	-		
**22**	**43**	C028776	M	64	Positive	Mild	Positive	Positive
	**44**	C028777	F	60	Negative	-		
**23**	**45**	C028778	M	37	Positive	Mild	Positive	Positive
	**46**	C028779	F	29	Negative	-		
**24**	**47**	C028780	M	49	Positive	Mild	Positive	Positive
	**48**	C028781	F	51	Negative	-		
**25**	**49**	C028782	M	56	Positive	Moderate	Positive	Positive
	**50**	C028783	F	55	Negative	-		
**26**	**51**	C028791	F	25	Positive	Mild	Positive	Positive
	**52**	C028792	M	24	Negative	-		
**27**	**53**	C028799	F	55	Positive	Moderate	Positive	Positive
	**54**	C028800	M	53	Negative	-		
**28**	**55**	C028806	M	43	Positive	Mild	Negative	Positive
	**56**	C028807	F	47	Negative	-		
**29**	**57**	C028812	F	36	Positive	Mild	Positive	Positive
	**58**	C028813	M	38	Negative	-		
**30**	**59**	C028814	F	33	Positive	Mild	Positive	Positive
	**60**	C028815	M	35	Negative	-		
**31**	**61**	C028825	M	64	Positive	Mild	Positive	Positive
	**62**	C028826	F	58	Negative	-		

**32**	**63**	C028827	F	26	Positive	Mild	Positive	Positive
	**64**	C028828	M	25	Negative	-		
**33**	**65**	C028838	M	49	Positive	Mild	Positive	Positive
	**66**	C028839	F	47	Negative	-		
**34**	**67**	C028840	M	82	Positive	Mild	Positive	Positive
	**68**	C028841	F	79	Negative	-		
**35**	**69**	C028848	M	33	Positive	Mild	Positive	Positive
	**70**	C028849	F	30	Negative	-		
**36**	**71**	C028852	M	58	Positive	Mild	Negative	Positive
	**72**	C028853	F	54	Negative	-		
**37**	**73**	C028856	M	46	Positive	Moderate	Positive	Positive
	**74**	C028857	F	40	Negative	-		
**38**	**75**	C028863	M	37	Positive	Mild	Negative	Positive
	**76**	C028864	M	29	Negative	-		
**39**	**77**	C028865	M	45	Positive	Mild	Positive	Positive
	**78**	C028866	F	45	Negative	-		
**40**	**79**	C028869	M	32	Positive	Mild	**Negative**	**Negative**
	**80**	C028870	F	31	Negative	-		
**41**	**81**	C028871	M	62	Positive	Mild	Positive	Positive
	**82**	C028872	F	53	Negative	-		
**42**	**83**	C028873	F	40	Positive	Mild	**Negative**	**Negative**
	**84**	C028874	M	50	Negative	-		
**43**	**85**	C028882	M	40	Positive	Mild	Positive	Positive
	**86**	C028883	F	42	Negative	-		
**44**	**87**	C028885	F	44	Positive	Mild	**Negative**	**Negative**
	**88**	C028886	M	46	Negative	-		
**45**	**89**	C028887	F	42	Positive	Mild	Positive	Negative
	**90**	C028888	M	28	Negative	-		
**46**	**91**	C028890	F	46	Positive	Mild	**Negative**	**Negative**
	**92**	C028891	M	47	Negative	-		
**47**	**95**	C028894	M	38	Positive	Mild	**Negative**	**Negative**
	**96**	C028895	F	33	Negative	-		

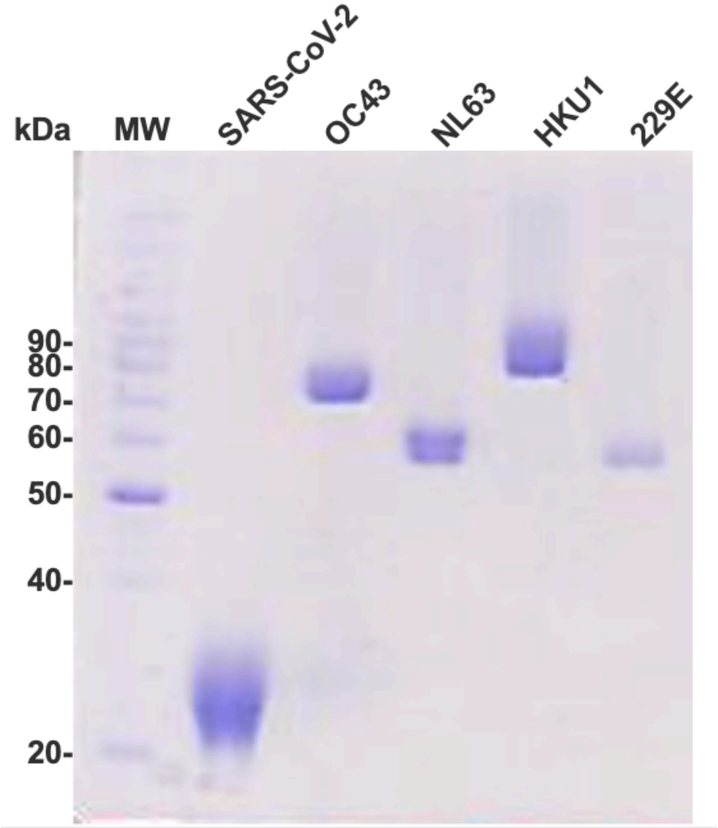

Supplementary Figure 1. RBDs from HCoV and SARS-CoV-2. RBDs were produced by transient transfection of Expi293 cells. Two µg of each protein were run on a polyacrylamide gel (SDS-PAGE 12%). Molecular weight in kDa is indicated on the left.

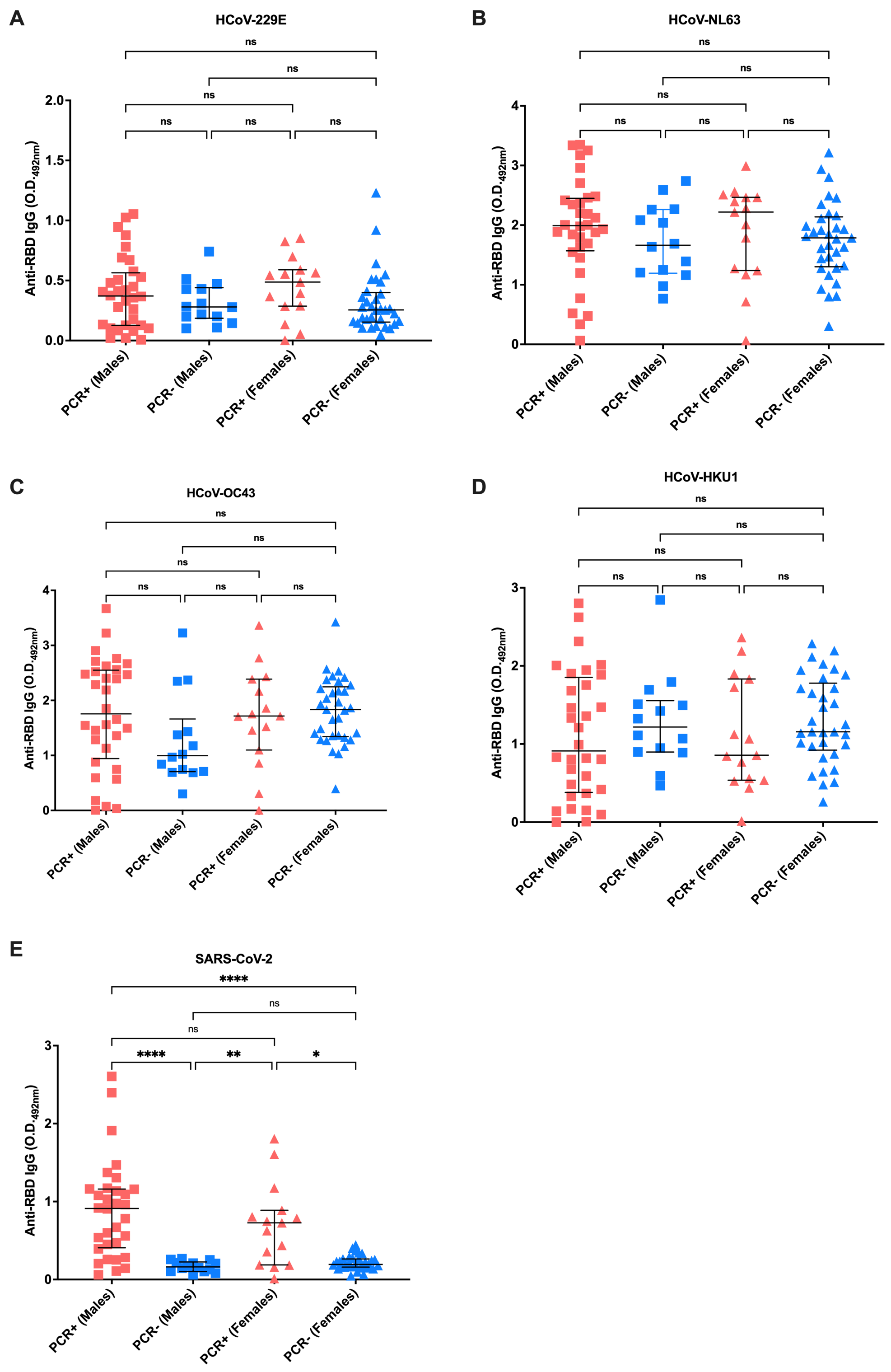

Supplementary Figure 2. Anti-RBD IgG serology profile of HCoV-229E, HCoV-OC43, HCoV-NL63 and HCoV HKU1 divided by gender. IgG response profile of PCR+ (males=32; females=15) or PCR- (males=14; females= 33) for SARS-CoV-2 against RBD proteins derived from alphacoronaviruses HCoV-229E (A) and HCoV-NL63 (B), betacoronaviruses HCoV-OC43 (C) and HCoV-HKU1 (D), and SARS-CoV-2 (E). For this assay, the subjects' plasma was used at a

concentration of 1:100, and the recombinant RBD in 2 μg/ml (100 ng/well). The test was read in the wavelength of 492 nm and each graph shows the O.D. obtained for the PCR+ (red) or PCR- (blue) individuals for SARS-CoV-2. The horizontal black lines represent the medians±interquartile intervals. Kruskal-Wallis followed by the Dunn's test. ns= not significant.
